# Supplementary material for: Compounded Effervescent Magnesium for Familial Hypomagnesemia: A Case Report
Source: Pharmaceuticals (Basel). 2023 May 24;16(6):785. doi: 10.3390/ph16060785 (PMC10305453; doi:10.3390/ph16060785)
Supplement: Supplementary file 1 [file pharmaceuticals-16-00785-s001.zip › pharmaceuticals-2348166-supplementary.pdf]

Compounded effervescent magnesium for familial hypomagnesemia: a case report.

Supplementary Material

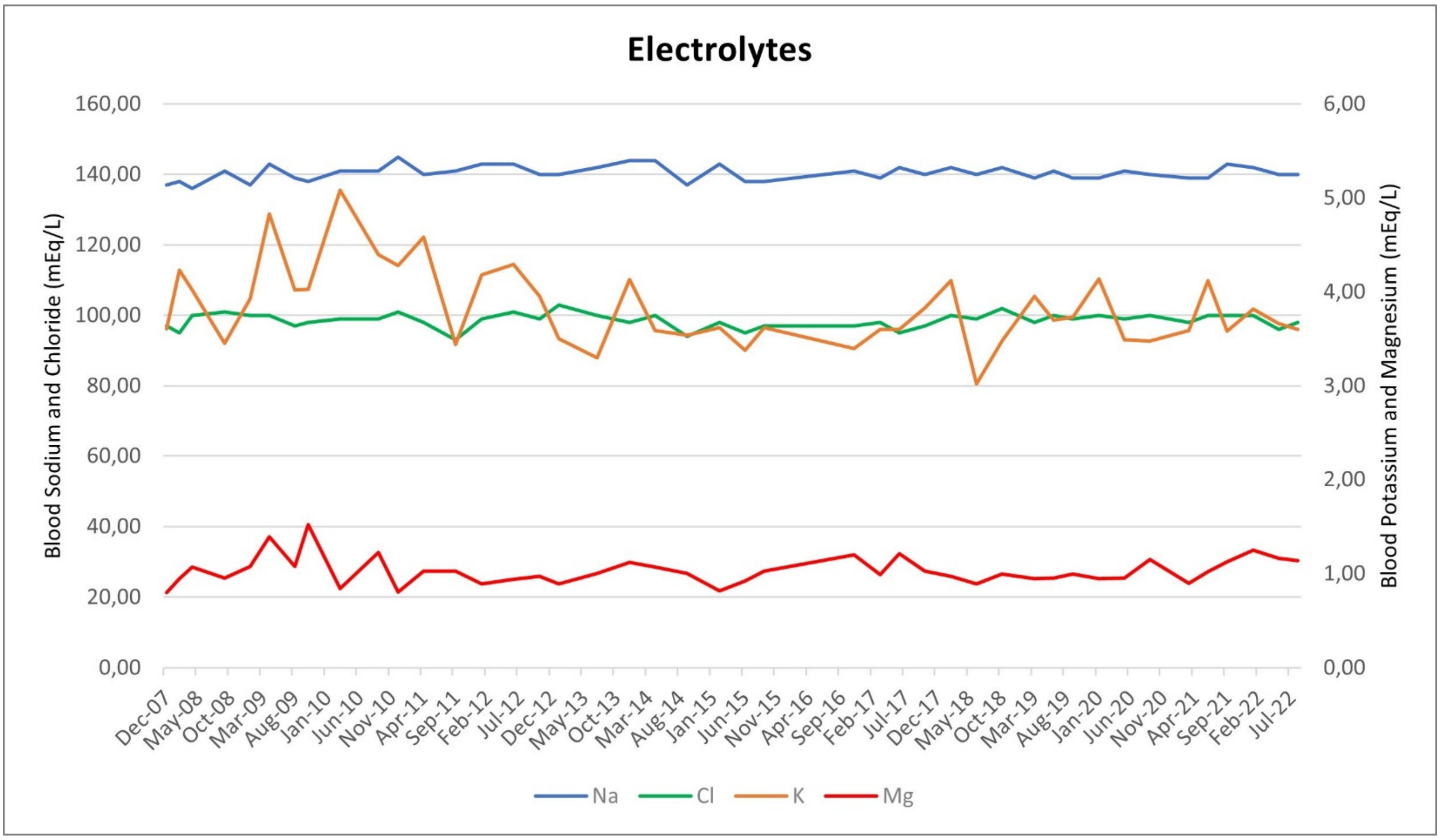

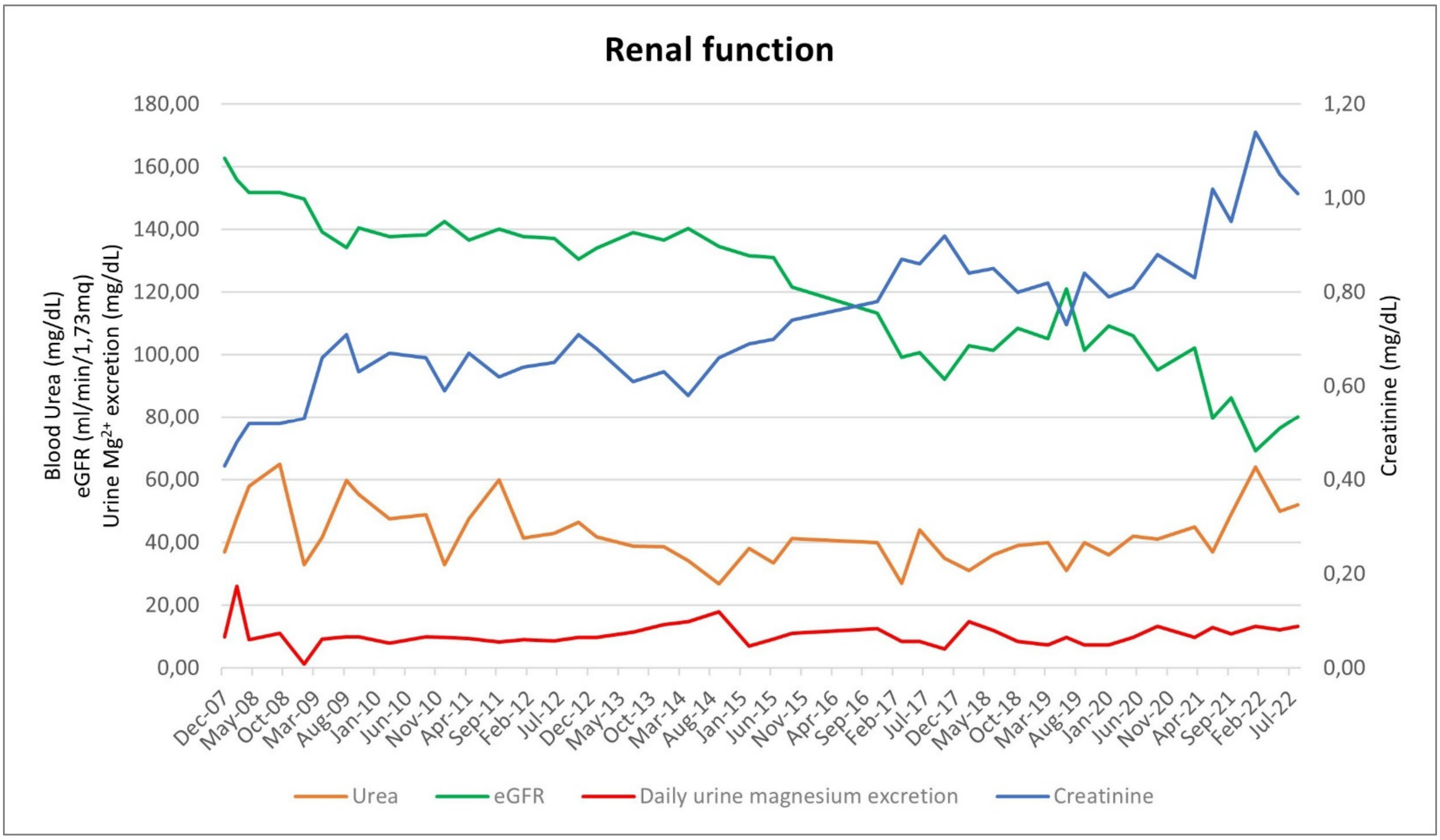

Figure S1: Blood electrolytes levels and renal function

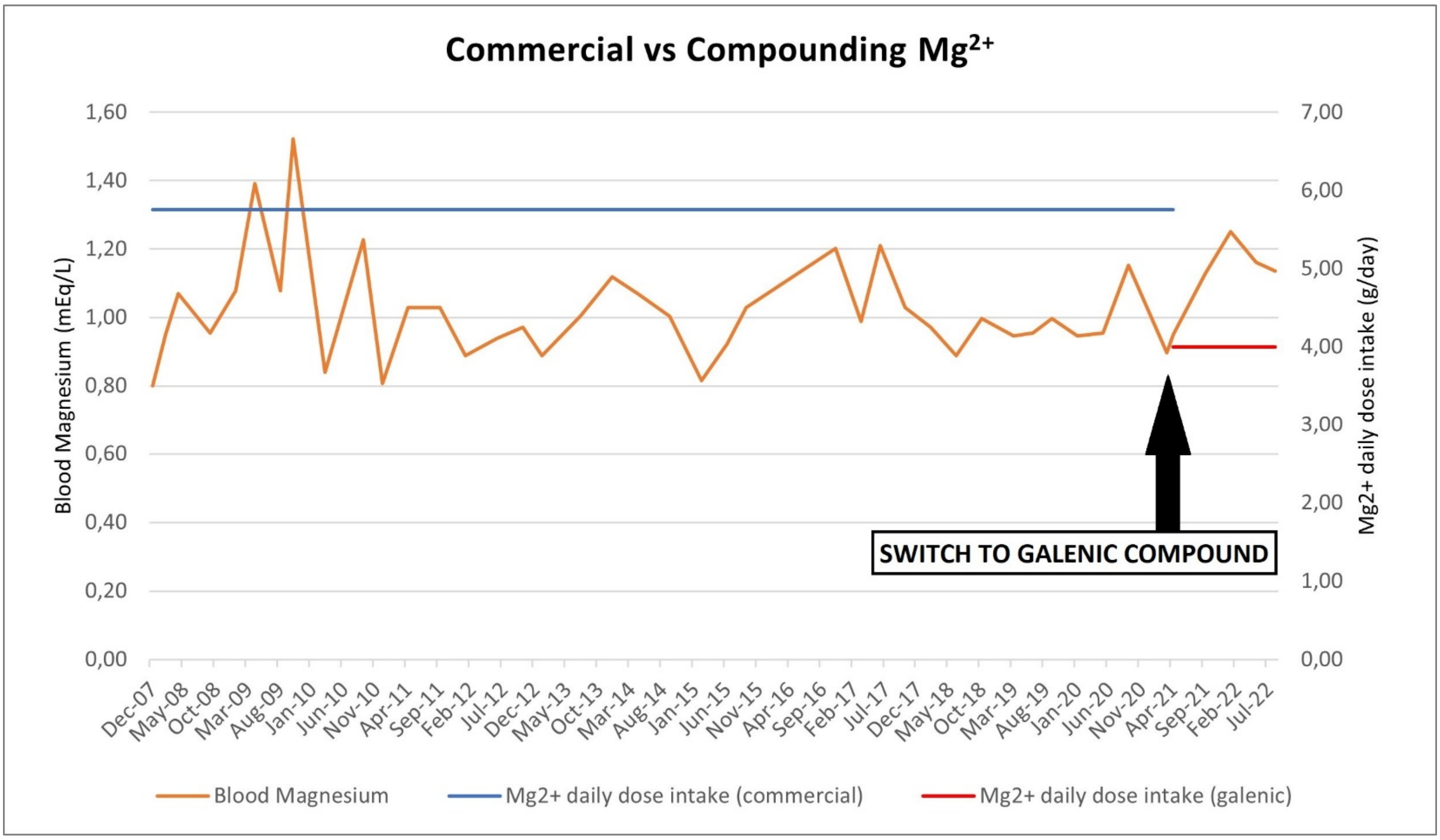

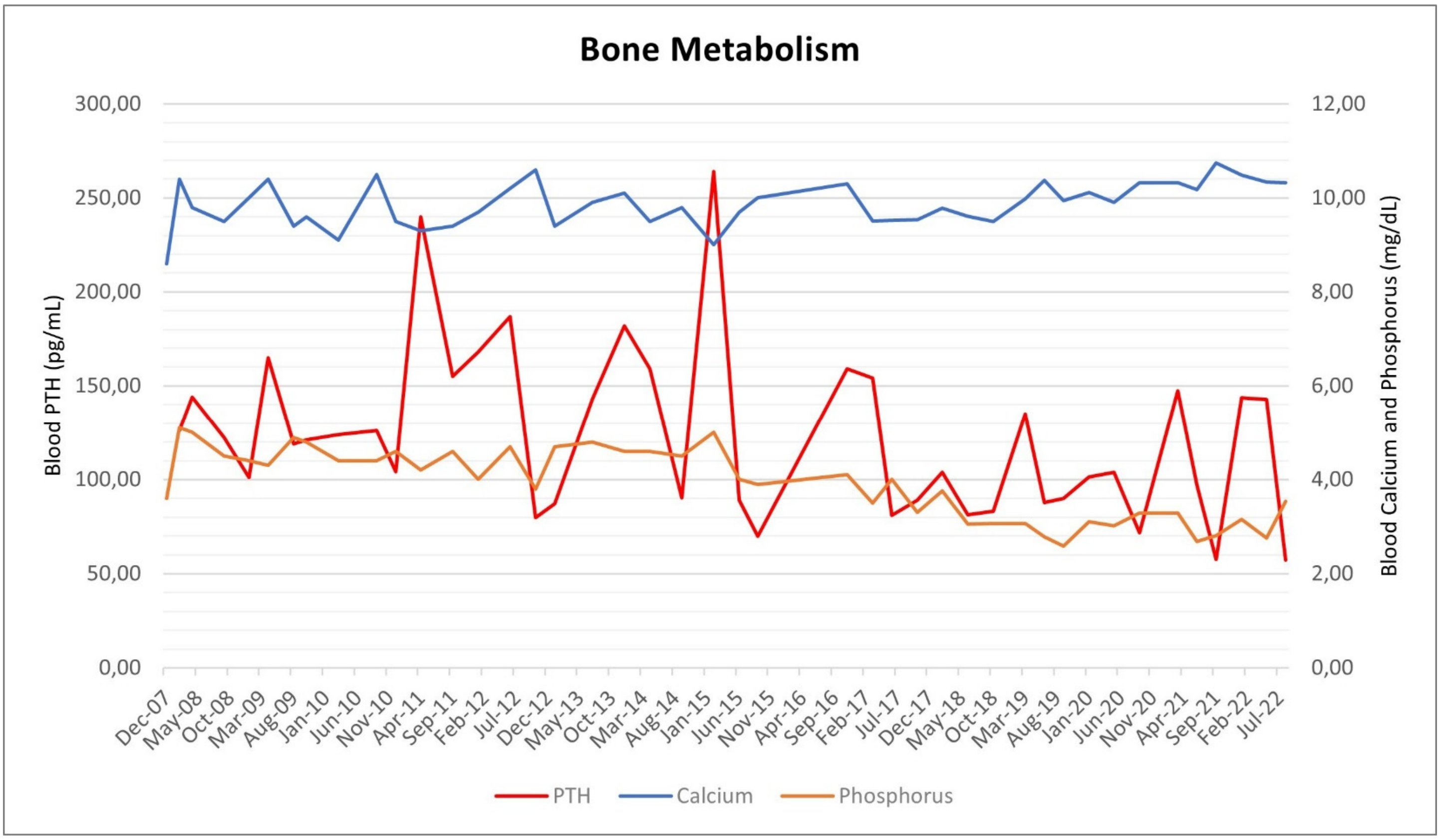

Figure S2: Blood magnesium levels related to daily magnesium doses and parameters of bone metabolism.

VISUAL ANALOG SCALE TEST **BEFORE** THE THERAPEUTIC SWITCH TO THE GALENIC COMPOUND

Parent

	Never	Almost never	Sometimes	Often	Almost always
Was it hard for your child to take the therapy?	0	1	2	3	X
Has your child forgotten to take the therapy?	0	1	X	3	4
Did your child dislike having to take the therapy all the time?	0	1	2	X	4

	Never	Almost never	Sometimes	Often	Almost always
Did you receive information about your child's general care?	0	1	2	X	4
Did the staff respond to your child's needs?	0	1	X	3	4
In general, was your child's health good?	0	X	2	3	4

Patient

	Never	Almost never	Sometimes	Often	Almost always
Was it hard for you to take the therapy?	0	1	2	3	X
Have you ever forgotten to take the therapy?	0	1	2	X	4
Did you dislike having to take the therapy all the time?	0	1	2	3	X

	Never	Almost never	Sometimes	Often	Almost always
Did you receive information about your general care?	0	1	2	X	4
Did the staff respond to your needs?	0	X	2	3	4
In general, was your health good?	0	X	2	3	4

VISUAL ANALOG SCALE TEST **AFTER** THE THERAPEUTIC SWITCH TO THE GALENIC COMPOUND

Parent

	Never	Almost never	Sometimes	Often	Almost always
Is it hard for your child to take the therapy?	X	1	2	3	4
Does your child forget to take the therapy?	X	1	2	3	4
Does your child dislike having to take the therapy all the time?	X	1	2	3	4

	Never	Almost never	Sometimes	Often	Almost always
Do you receive information about your child's general care?	0	1	2	3	X
Does the staff respond to your child's needs?	0	1	2	3	X
In general, is your child's health good?	0	1	2	X	4

Patient

	Never	Almost never	Sometimes	Often	Almost always
Is it hard for you take the therapy?	X	1	2	3	4
Do you forget to take the therapy?	X	1	2	3	4
Do you dislike taking the therapy all the time?	X	1	2	3	4

	Never	Almost never	Sometimes	Often	Almost always
Do you receive information about your general care?	0	1	2	3	X
Does the staff respond to your needs?	0	1	2	3	X
In general, is your health good?	0	1	2	3	X

Figure S3: VAS questionnaire submitted to the patient and parent BEFORE and AFTER the transition to galenic compound.
